# Enhancement of Ar Ion Flux on the Substrate by Heterogeneous Charge Transfer Collision of Ar Atom with He Ion in an Inductively Coupled Ar/He Plasma

**DOI:** 10.3390/ma16175746

**Published:** 2023-08-22

**Authors:** Inho Seong, Sijun Kim, Minsu Choi, Woobeen Lee, Wonnyoung Jeong, Chulhee Cho, Yebin You, Youngseok Lee, Youbin Seol, Shinjae You

**Affiliations:** 1 Applied Physics Lab for PLasma Engineering (APPLE), Department of Physics, Chungnam National University, Daejeon 34134, Republic of Koreaeub2nzeeno@naver.com (W.L.);; 2 Institute of Quantum Systems (IQS), Chungnam National University, Daejeon 34134, Republic of Korea

**Keywords:** ion flux, ion energy distribution function, He addition, charge transfer collision, heterogeneous charge transfer collision

## Abstract

The understanding of ion dynamics in plasma applications has received significant attention. In this study, we examined these effects between He and Ar species, focusing on the Ar ion flux on the substrate. To control heterogeneous collisions, we varied the He addition rate at fixed chamber pressure and the chamber pressure at fixed Ar/He ratio in an inductively coupled Ar/He plasma source. Throughout the experiments, we maintained an electron density in the bulk plasma and plasma potential as a constant value by adjusting the RF power and applying an additional DC bias to eliminate any disturbances caused by the plasma. Our findings revealed that the addition of He enhances the Ar ion flux, despite a decrease in the Ar ion density at the plasma–sheath boundary due to the presence of He ions. Moreover, we found that this enhancement becomes more prominent with increasing pressure at a fixed He addition rate. These results suggest that the heterogeneous charge transfer collision between Ar atoms and He ions in the sheath region creates additional Ar ions, ultimately leading to an increased Ar ion flux on the substrate. This finding highlights the potential of utilizing heterogeneous charge transfer collisions to enhance ion flux in plasma processing, without the employment of additional equipment.

## 1. Introduction

Plasma, also known as the fourth state of matter, is an ionized gas that exhibits collective behavior and electrically quasi-neutral characteristics [1]. It consists of charged particles such as electrons, positive and negative ions, as well as neutrals and radicals. This composition makes plasma chemically reactive and physically energetic [2]. Radical species primarily govern the chemical reactions both within the bulk plasma and on the material surfaces exposed to plasma. Positive ions, on the other hand, play a crucial role in transferring physical energy to the material surfaces, thereby inducing surface activation and sputtering. Due to its unique properties, plasma has found widespread application in high-end technologies such as semiconductor/display fabrication [3], spacecrafts [4,5], agriculture [6,7], and medicine [8,9]. Notably, plasma has garnered significant attention in next-generation plasma-etching technologies for semiconductor fabrication, including plasma-enhanced atomic layer etching [10], high-aspect ratio etching [11], and cryogenic etching [12,13].

Radical species play a dominant role in surface chemistry processes such as etching, polymerization, and oxidation on material surfaces. These radicals are primarily generated through dissociative electron impact collisions between electrons in the plasma and feedstock gases. For instance, in the plasma etching of silicon (Si) and silicon dioxide (SiO${}_{2}$), radicals originate from feedstock gases like C${}_{4}$F${}_{8}$, CF${}_{4}$, SF${}_{6}$, and CHF${}_{3}$. They serve as the main etchant, removing Si by producing volatile byproducts like SiF${}_{4}$ [14,15]. Radicals also act indirectly as etchants, forming mixed layers (SiO${}_{2}$-fluorocarbon) on the SiO${}_{2}$ surface. These mixed layers reduce bonding energy, enabling subsequent removal through positive ion bombardment, which produces byproducts like SiF${}_{4}$ and CO [16]. Additionally, radicals can act as inhibitors of etching by forming passivation layers, which protect non-target materials from etching. For instance, fluorocarbon (FC) species can leave a carbon layer on the underlying Si surface, offering protection against ion bombardment. The precise measurement of radical species necessitates the use of various instruments and techniques, including actinometry with an optical emission spectrometer [17,18] and appearance potential method with a mass spectrometer [19]. Furthermore, since these radicals are produced through dissociative electron impact collisions, it is essential to measure electron parameters to control radical species. Various electron parameter diagnostics applicable in plasma processing have been developed, including microwave probes [20,21], emissive probes [22], and voltage/current probes [23].

Understanding positive ion dynamics, including ion bombardment effects and transport in the sheath toward material surfaces, is crucial as positive ion species predominantly influence physical processes on surfaces [24]. For instance, ions transfer their kinetic energy to material surfaces, and the effects vary depending on the ion energy range [25]. Ion bombardment of the material surface leads to collision cascades, resulting in processes such as gas desorption, chemical sputtering, physical sputtering, and implantation [26]. At low ion energies, ion bombardment can be approximated as binary collisions between incident ions and target atoms [27], leading to gas desorption by breaking the physisorption bonds, such as Van der Waals force, formed between gases and surface atoms [28]. As ion energy increases, the ion bombardment alters from binary collisions to collision cascades, causing chemical and physical sputtering through successive collisions. Chemical sputtering is particularly significant in plasma etching mechanisms, as it removes mixed layers, as mentioned earlier [2]. Furthermore, at ion energies exceeding kiloelectron volts (keV), ion bombardment is utilized in ion implantation processes [29]. High-energy ions penetrate the target material through collisions with sub-surface atom layers, eventually resulting in implantation. In addition to kinetic energy transfer, ions also induce secondary electron emission, which is a primary process for initiating and sustaining glow discharge, a commonly used region in plasma processing [14].

In addition to the effects of ion bombardment, the transport of ions in the sheath has received considerable attention as it determines the ion energy and incident angle distributions on the material surface [30]. These factors, in turn, influence the aforementioned effects such as etching rate [31], sputtering yield [32], and secondary electron emission coefficient [33]. The sheath refers to the positive space charge region between the plasma and the material facing the plasma. Due to the electric field generated by positive space charges, ions can be accelerated toward the material surface. In a collisionless sheath where the collision mean free path is larger than the sheath width, the ion flux–energy distribution function (IFEDF) is influenced by the relationship between the ion transit time and the sheath oscillation time [34]. In a collisional sheath, the IFEDF strongly depends on collision characteristics, such as elastic and charge transfer collisions. Elastic collisions shift the IFEDF to lower energies and broaden the ion angle distribution. In highly collisional sheaths, the IFEDF approaches a Maxwellian distribution due to elastic collisions. Charge transfer collisions between same species, for instance Ar neutral and Ar${}^{+}$ ion, can produce multiple peaks in the IFEDF at low ion energies [35] and also contribute to neutral gas heating [36].

On the contrary, for the influence of heterogeneous collisions on ion dynamics in the sheath, which mean the collisions between different species, only a few studies have been conducted on cross-sections [37,38] and IFEDF [39]. Therefore, in this study, we investigate the effects of heterogeneous collisions between He and Ar species, particularly focusing on the Ar ion flux on the substrate in an inductively coupled Ar/He plasma.

This paper is organized as follows. Section 2 describes the inductively coupled plasma source, as well as the measurement methodologies of plasma parameters and IFEDFs. In the subsequent section, we present the experimental results, which demonstrate the constant plasma parameters and the behavior of the IFEDFs under varying He addition rates and chamber pressures. In Section 4, we discuss the cause of the Ar ion flux enhancement, focusing on the effect of heterogeneous collisions. Finally, we conclude this paper in the last section.

## 2. Methods

### 2.1. Plasma Generation

**Figure 1:**
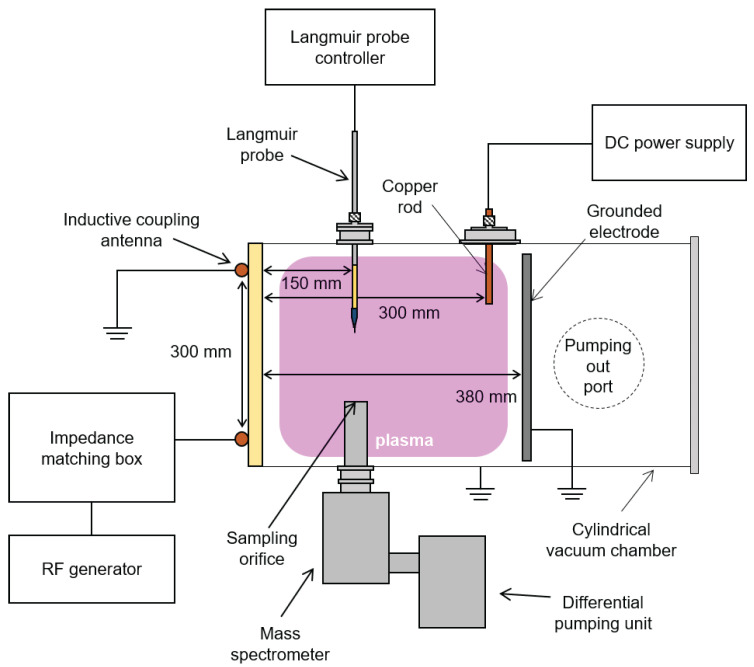
Schematic diagram of experiment setup.

We employed an inductively coupled plasma (ICP) source, the details of which have been comprehensively described elsewhere [40]. In this section, we provide a brief overview of the system. Figure 1 illustrates a cross-sectional view of the ICP source. Ar and He gases are introduced into the vacuum chamber through mass flow controllers (MFC, LineTech Inc., Deajeon, Republic of Korea). The turbo-molecular pump (ATP400,Alcatel Vacuum Technology, France) and the rotary pump (DS102, Agilent Inc., Santa Clara, CA, USA) evacuate the vacuum chamber through the pumping port to maintain the desired chamber pressure, which is measured by the vacuum gauge (Baratron, MKS Instrument Inc., Andover, MA, USA). The radio-frequency (RF) generator (RFPG-600AI, RFPMT, Republic of Korea) supplies 13.56 MHz RF power to the inductively coupling antenna, which has a diameter of 300 mm, via the L-type impedance matching box, thus creating the plasma.

We maintained the electron density and plasma potential at fixed values to mitigate any disturbances by plasma-induced effects such as ion flux and energy variations by plasma density and potential fluctuations, respectively. To control these parameters, we controlled both the RF power and the applied direct current (DC) bias voltage (TDP-2001B, Toyotech, Incheon, Republic of Korea) on the copper rod depicted in Figure 1. Typically, the electron density is determined by the RF power, while the plasma potential depends on the DC bias voltage. The electron density variation is a result of the increased ionization rate due to enhanced electron heating, whereas the plasma potential dependence stems from the suppression of electron extraction from the plasma to the copper rod when the DC bias voltage exceeds the plasma potential.

### 2.2. He Addition and Pressure Variation Method

For the He addition experiment, the displayed helium flow rate from the MFC controller is not accurate because the MFC used for He gas injection is optimized for Ar gas injection. Alternatively, we utilized the vacuum gauge to accurately measure the partial pressures of Ar and He gases, enabling the precise control of the small fraction of He addition ratio, typically a few percent. During the IFEDF measurement, the chamber pressure was set to 1.3 Pa (10 mTorr). However, at such low pressure, the He partial pressure fell below 0.1 Pa (1 mTorr), which was below the minimum measurement range of the vacuum gauge. To overcome this limitation, we employed an alternative method described below.

Initially, Ar gas was introduced to establish a chamber pressure of 13.3 Pa (100 mTorr). Then, He gas was injected to increase the chamber pressure to approximately 0.3 Pa (2 mTorr), resulting in the total pressure of 13.6 Pa (102 mTorr). The gate valve, located beneath the pumping outlet shown in Figure 1, was closed to reduce the chamber pressure to 1.3 Pa (10 mTorr). Consequently, the He addition ratio was calculated to be 1.96% (=(0.27 Pa/13.6 Pa) × 100(%)). By employing this approach, we successfully introduced He gas ranging from 0 Pa to 1.3 Pa (10 mTorr), corresponding to He addition ratios of 0% to 9.09% at a chamber pressure of 1.3 Pa (10 mTorr).

Regarding the pressure variation experiment, we adjusted the open ratio of the gate valve while maintaining a fixed gas mixture of Ar/He at a ratio of 15 to 5.

### 2.3. Plasma Diagnostics

To measure electron density ($n_{e}$) and plasma potential ($V_{p}$), we employed a Langmuir probe capable of measuring $n_{e}$, $V_{p}$, electron temperature, and the electron energy probability function ($f_{\mathrm{EEPF}}$) as shown in Figure 1. For accurate measurements, we implemented two RF choke filters (13.56 MHz and 27.12 MHz band stop filters) to eliminate the voltage drop of RF plasma potential on the probe tip. The probe assembly consisted of a ceramic tube enclosing the filters and a stainless steel tube holding the ceramic tube. The probe tip itself was made of tungsten with a diameter of 0.25 mm and covered by a ceramic tube with a diameter of 0.5 mm. The exposed length of the probe tip was 1.5 mm.

**Figure A1:**
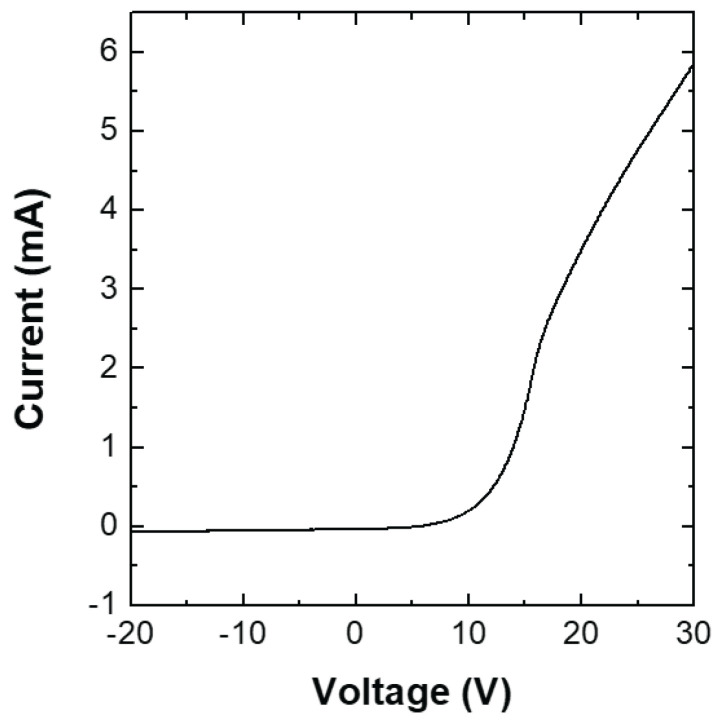
Current–voltage (I-V) curve of the Langmuir probe at RF Power of 400 W, chamber pressure of 0.7 Pa (5 mTorr), and Ar/He ratio (Ar:He = 15:5).

To sweep the voltage (V) across the Langmuir probe and measure the resulting current (I), we employed a commercial control system (Wise Probe System, P&A Solutions, Seoul, Republic of Korea) and accompanying software (WiseSLP, P&A Solutions, Seoul, Republic of Korea). Since the Langmuir probe method is widely used [22,40], we provide a brief description here. We swept probe voltages ranging from −20 V to 30 V with averaging of 25 iterations. The current–voltage (I-V) curve measured at specific conditions is represented in Figure A1. The electron density ($n_{e}$) is obtained by integrating the $f_{\mathrm{EEPF}}$ over the electron energy (ξ), expressed as:

(1) $$n_{e}=\int_{0}^{\infty }f_{\mathrm{EEPF}}\left(\xi \right)d\xi .$$

The plasma potential ($V_{p}$) is determined at the “knee” of the I-V curve, which corresponds to the point where the first derivative of the I-V curve becomes the maximum peak:

(2) $$\frac{d^{2}I}{dV^{2}}{|}_{V=Vp}=0.$$

### 2.4. Ion Flux Energy Distribution Function (IFEDF) Measurement

As shown in Figure 1, we adopted a quadrupole mass spectrometer (PSM, Hiden Analytical Inc., Warrington, Cheshire, UK) consisting of an ionizer, Bessel box (ion energy filter), ion optics, and triple quadrupole mass filters. Here is a brief overview of the instrument’s working principle. In the ion measurement mode, the ionizer is deactivated, allowing only plasma ions to directly enter the Bessel box. Within the Bessel box, ions with only specific energy can pass through and proceed to the ion optics. The ion optics then focus the ions towards the entrance of the mass filter vessel, which contains triple quadrupole mass filters. Only ions with specific masses can pass through the mass filter vessel and ultimately reach the detector. By sweeping the voltage of the Bessel box, we can obtain the IFEDF, as demonstrated in a previous study by the authors [41]. In the present study, we set the ion filtering mass to 40 amu (atomic mass unit) for the detection of Ar ions, enabling the measurement of the IFEDF of Ar ions.

To calculate the total Ar ion flux ($\Gamma_{{\mathrm{Ar}}^{+}}$), we integrated the IFEDF (g(E)) over ion energy (*E*) using the following equation:

(3) $$\Gamma_{{\mathrm{Ar}}^{+}}=\int_{0}^{\infty }g\left(E\right)dE$$

The sampling orifice of the mass spectrometer has a diameter of 100 μm. To maintain the pressure within the mass filter vessel below 1.3 × 10${}^{-6}$ Pa (10${}^{-8}$ Torr), we employed a differential pumping unit comprising a turbo-molecular pump (nEXT 300D, Edwards, Burgess Hill, UK) and a rotary pump (DUO 3, Pfeiffer Vacuum Inc, Nashua, NH, USA). The pressure in the mass filter vessel was measured using a full-range gauge (D-35614 Assair, Pfeiffer Vacuum Inc, Nashua, NH, USA).

## 3. Results

### 3.1. He Addition Effect at Fixed Chamber Pressure

**Figure 2:**
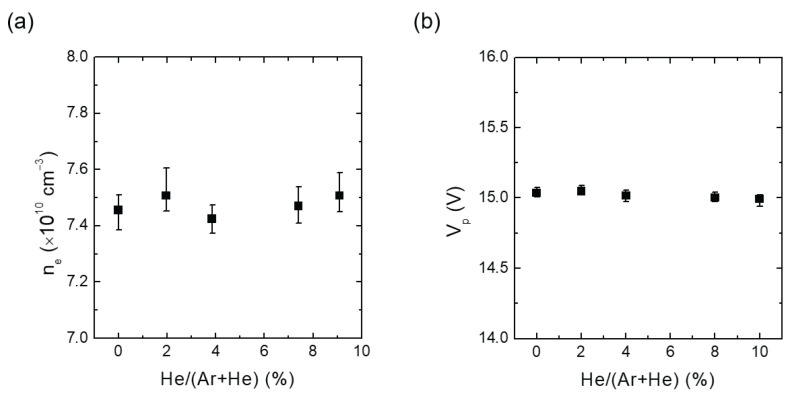
(**a**) Measured electron density and (**b**) plasma potential against He addition ratio (He/(Ar+He)) at a constant chamber pressure of 1.3 Pa (10 mTorr).

To investigate the effect of He addition at a fixed chamber pressure, He gas was injected into the chamber filled with pure Argon gas to induce heterogeneous collisions. The IFEDF was measured as a function of the He addition ratio (He/(Ar+He)) at a constant chamber pressure. Figure 2a,b show the measured $n_{e}$ and $V_{p}$, respectively, for increasing He addition ratios ranging from 0% to 9.09% at a chamber pressure of 1.3 Pa (10 mTorr). While the He addition ratio increased, $n_{e}$ and $V_{p}$ were maintained at fixed values with decreasing RF power and increasing DC bias voltage. Specifically, $n_{e}$ was maintained between 7.42 × 10${}^{10}$ cm${}^{-3}$ and 7.51 × 10${}^{10}$ cm${}^{-3}$, and $V_{p}$ remained in the range from 15.00 V to 15.05 V. The variations in $n_{e}$ and $V_{p}$ were 1.2% and 0.3%, respectively.

**Figure 3:**
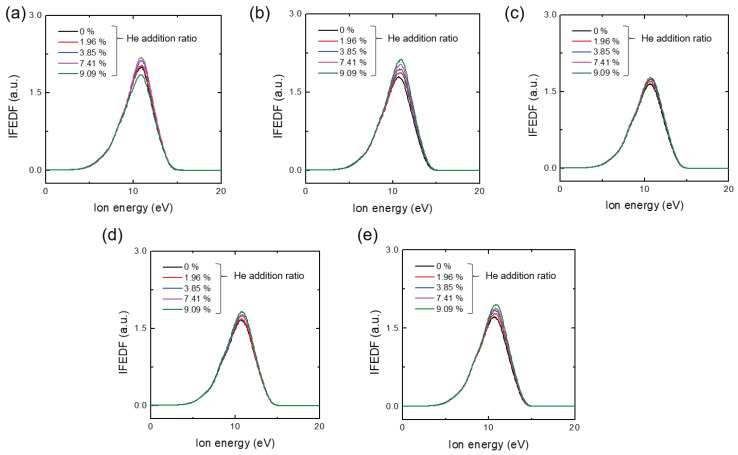
Ion flux-energy distribution function (IFEDF) with various additive He ratio at a constant chamber pressure of 1.3 Pa (10 mTorr) at (**a**) 1st, (**b**) 2nd, (**c**) 3rd, (**d**) 4th, and (**e**) 5th trials. The pressure of the mass spectrometer is 1.9 × 10${}^{-4}$ Pa.

**Figure 4:**
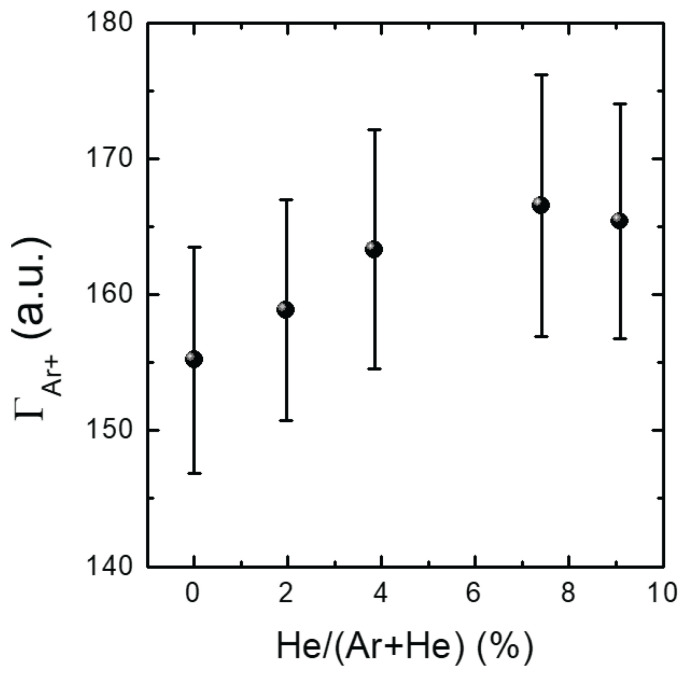
Ion flux ($\Gamma_{{\mathrm{Ar}}^{+}}$) against additive He ratio at a constant chamber pressure of 1.3 Pa (10 mTorr).

Figure 3a–e show the measured Ar IFEDF over ion energy for various He addition ratios, obtained through five trials. Here, multiple measurements were conducted to ensure measurement consistency due to signal degradation of the mass spectrometer over time (see the IFEDF values from Figure 3a–e). All IFEDFs exhibited a single-peak shape with a peak energy of 10.8 eV. With the exception of the first trial, their peaks consistently increased monotonically with increasing He addition ratio, while the peak energy remained constant. This indicates an increased number of ions near the peak energy, which can affect the $\Gamma_{{\mathrm{Ar}}^{+}}$. Figure 4 presents the averaged $\Gamma_{{\mathrm{Ar}}^{+}}$ over the five trials as a function of the He addition ratio. The large discrepancy observed can be attributed to signal degradation of the mass spectrometer, as indicated in Figure 3. Nevertheless, it is evident that $\Gamma_{{\mathrm{Ar}}^{+}}$ monotonically increased with the He addition ratio, except at the ratio of 9.09%. Notably, the increase in $\Gamma_{{\mathrm{Ar}}^{+}}$ from the minimum to the maximum value in Figure 4 was approximately 7.5%. This increase was much larger than the perturbations observed in the plasma parameters shown in Figure 2, indicating that the increase in Ar ion flux on the substrate was primarily caused by the small fraction of He addition.

### 3.2. Chamber Pressure Variation Effect at Fixed Ar/He Ratio

**Figure 5:**
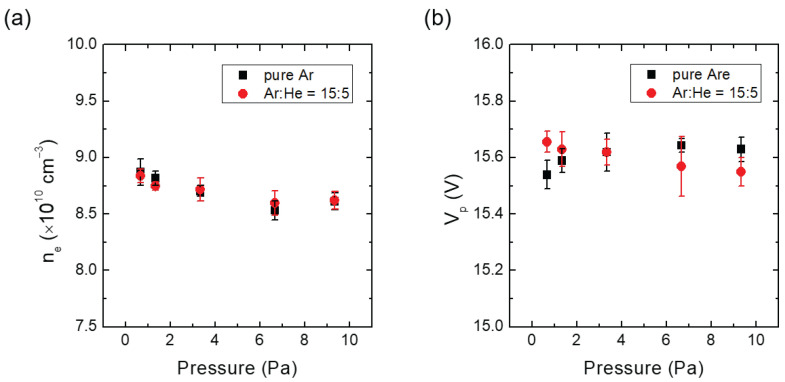
(**a**) Measured electron density and (**b**) plasma potential at pure Ar and fixed Ar/He ratio (Ar:He = 15:5) against chamber pressure.

To examine the effects of chamber pressure variation at a fixed Ar/He ratio, the IFEDF was investigated as the chamber pressure increased. Additionally, the IFEDF was examined in a pure Ar environment with the same chamber pressure variations, serving as a reference to distinguish between homogeneous and heterogeneous collision effects within the sheath. Figure 5a,b illustrate $n_{e}$ and $V_{p}$, respectively, for various chamber pressures ranging from 0.7 Pa (5 mTorr) to 9.3 Pa (70 mTorr) in both the pure Ar and Ar/He environments with a fixed Ar/He ratio of 15 to 5. As shown in Figure 5, both $n_{e}$ and $V_{p}$ remained constant across the investigated chamber pressures, with $n_{e}$ ranging from 8.53 × 10${}^{10}$ cm${}^{-3}$ to 8.87 × 10${}^{10}$ cm${}^{-3}$ and $V_{p}$ ranging from 15.54 V to 15.65 V. The variations in $n_{e}$ and $V_{p}$ were approximately 4% and 0.7%, respectively. To fix $n_{e}$ and $V_{p}$, we lowered the RF power until 6.7 Pa (50 mTorr) and then, increased it at 9.3 Pa (70 mTorr), whereas we continuously raised the $V_{\mathrm{DC}}$ from 0.7 to 9.3 Pa (5 to 70 mTorr) during pressure variations at the pure Ar condition. Similarly, we lowered the RF power until 3.3 Pa (25 mTorr) and then, increased it after the pressure condition of 3.3 Pa (25 mTorr), whereas we continuously raised the $V_{\mathrm{DC}}$ from 0.67 to 9.3 Pa (5 to 70 mTorr) during pressure variations at the Ar/He condition.

**Figure 6:**
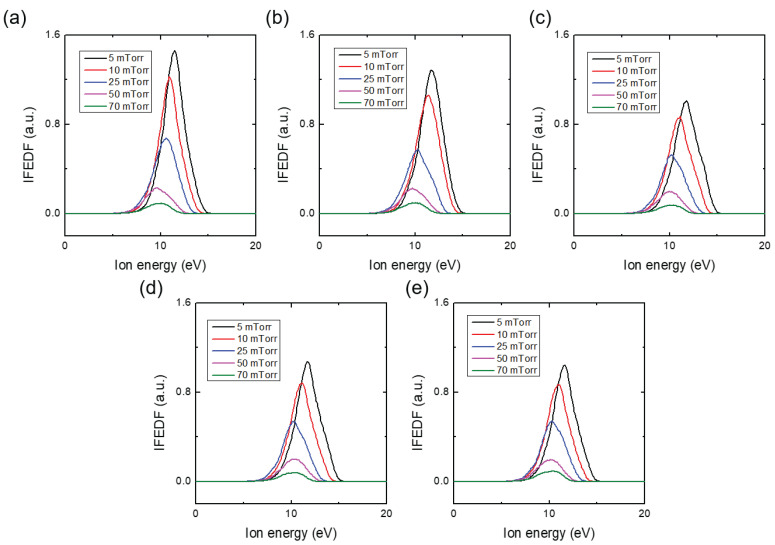
Ion flux–energy distribution function (IFEDF) with various chamber pressures with fixed Ar/He ratio (Ar:He = 15:5) at (**a**) 1st, (**b**) 2nd, (**c**) 3rd, (**d**) 4th, and (**e**) 5th trials. At the chamber pressure of 9.3 Pa, the pressure of the mass spectrometer is 5.2 × 10${}^{-4}$ Pa.

**Figure 7:**
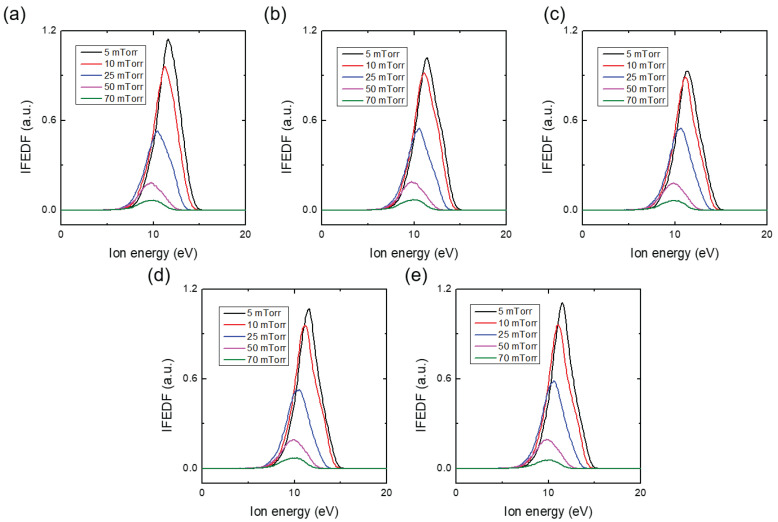
Ion flux-energy distribution function (IFEDF) with various chamber pressures in pure Ar environment at (**a**) 1st, (**b**) 2nd, (**c**) 3rd, (**d**) 4th, and (**e**) 5th trials. At the chamber pressure of 9.3 Pa, the pressure of the mass spectrometer is 5.2 × 10${}^{-4}$ Pa.

**Figure 8:**
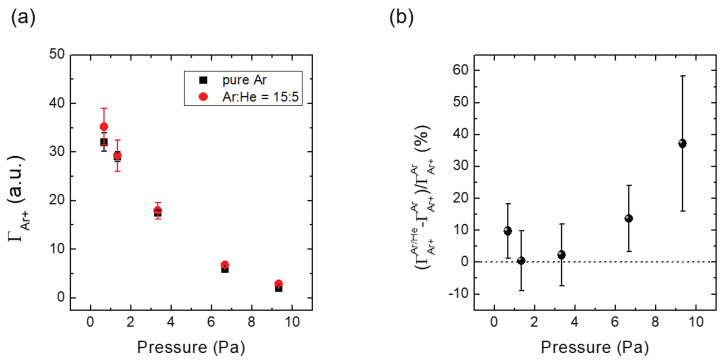
(**a**) Ion flux ($\Gamma_{{\mathrm{Ar}}^{+}}$) over chamber pressure at pure Ar and fixed Ar/He ratio (Ar:He = 15:5) (**b**) normalized Ar${}^{+}$ flux against chamber pressure.

Figure 6 displays the IFEDF over ion energy for various chamber pressures in the Ar/He environment, acquired through five trials. Increasing the chamber pressure resulted in a significant decrease in the IFEDF and a shift towards lower ion energies for the peak. Figure 7 presents the IFEDF over ion energy in the pure Ar environment, also showing a decrease in the IFEDF and a shift towards lower ion energies for the peak. These results indicate that the decrease and peak shift originated from enhanced collisions within the sheath as the chamber pressure increased [14]. Figure 8a illustrates the averaged $\Gamma_{{\mathrm{Ar}}^{+}}$ over five trials as a function of the chamber pressure in both the pure Ar and Ar/He environments. In both cases, $\Gamma_{{\mathrm{Ar}}^{+}}$ monotonically decreased with increasing pressure. Notably, the $\Gamma_{{\mathrm{Ar}}^{+}}$ was higher in the Ar/He case compared to the pure Ar case except the 0.7 Pa (5 mTorr) condition. Figure 8b, which represents the enhancement ratio of the $\Gamma_{{\mathrm{Ar}}^{+}}$ in the Ar/He case compared to the pure Ar case, clearly shows the enhancement of the $\Gamma_{{\mathrm{Ar}}^{+}}$ in the Ar/He case. With the exception of the 0.7 Pa (5 mTorr) pressure condition, the enhancement ratio increased up to nearly 40% with increasing pressure, indicating additional creation of Ar ions within the sheath through heterogeneous collisions between Ar and He species, which is further discussed in the next section. This 40% increase is significantly larger than the variations observed in the plasma parameters shown in Figure 5, providing further evidence that the increase in Ar ion flux on the substrate was indeed caused by heterogeneous collisions.

## 4. Discussion

**Figure 9:**
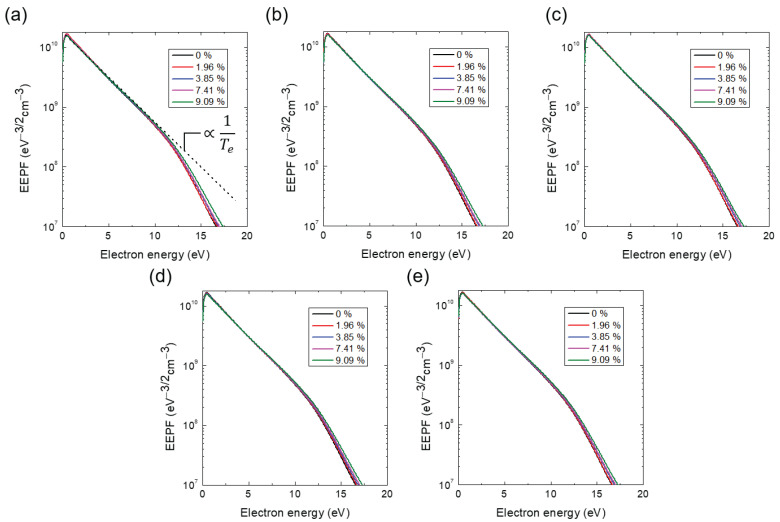
Electron energy probability function (EEPF) with various additive He ratio at chamber pressure of 1.3 Pa (10 mTorr) at (**a**) 1st, (**b**) 2nd, (**c**) 3rd, (**d**) 4th, and (**e**) 5th trials.

The ion flux at the plasma–sheath boundary (PSB), $\Gamma_{{\mathrm{Ar}}^{+}}^{\mathrm{PSB}}$, can be described by the equation:

(4) $$\Gamma_{{\mathrm{Ar}}^{+}}^{\mathrm{PSB}}=n_{{\mathrm{Ar}}^{+}}u_{B},$$

where $n_{{\mathrm{Ar}}^{+}}$ is the Ar ion density, $u_{B}$ represents the Bohm velocity ($\sqrt{eT_{e}/M_{\mathrm{Ar}}}$), *e* is the elementary charge, $T_{e}$ is the electron temperature, and $M_{\mathrm{Ar}}$ is the mass of the Ar ion. Here, we assumed that each ion species have their own Bohm velocity [14]. In an Ar/He plasma, the quasi-neutrality condition states that the total density of positively charged species is equal to the total density of negatively charged species, and it can be expressed as:

(5) $$n_{e}=n_{{\mathrm{Ar}}^{+}}+n_{{\mathrm{He}}^{+}},$$

where $n_{{\mathrm{He}}^{+}}$ represents the He ion density. In all experiments shown in Figure 2a, $n_{e}$ was maintained as a fixed value. According to Equation (5), the addition of He ions should result in a decrease in $n_{{\mathrm{Ar}}^{+}}$ due to the increase in $n_{{\mathrm{He}}^{+}}$. Additionally, the Bohm velocity $u_{B}$ in Equation (4) is a function of $T_{e}$. Figure 9a–e show the measured $f_{\mathrm{EEPF}}$ in which, the slope of the $f_{\mathrm{EEPF}}$ is inversely proportional to the $T_{e}$ as shown in Figure 9a. All $f_{\mathrm{EEPF}}$s in Figure 9 indicate a slight increase in $T_{e}$. Since the change in $T_{e}$ is negligible, $T_{e}$ can be assumed to be constant. Therefore, based on Equation (4), we would expect $\Gamma_{{\mathrm{Ar}}^{+}}^{\mathrm{PSB}}$ to decrease with increasing He addition ratio and, thus, the measured $\Gamma_{{\mathrm{Ar}}^{+}}$ is also expected to drop. However, Figure 4 reveals an increase in $\Gamma_{{\mathrm{Ar}}^{+}}$ on the substrate, suggesting the additional creation of Ar ions in the sheath. This phenomenon can be attributed to heterogeneous collisions between Ar and He species, such as ion-atom ionization and charge transfer collisions.

The heterogeneous ion-atom ionization between an Ar atom and a He ion is expressed as:

(6) $$\mathrm{Ar}+{\mathrm{He}}^{+}\overset{}{\to }{\mathrm{Ar}}^{+}+{\mathrm{He}}^{+}+e.$$

Typically, ions can transfer only a small fraction of their kinetic energy, approximately ∼$2m_{e}/M_{i}$, to a valence electron in an atom, where $m_{e}$ and $M_{i}$ are the mass of the electron and the ion, respectively. This ion-atom ionization process becomes significant for high-energy ions (>100 eV) [14]. Considering the IFEDF shown in Figure 3a–e, most ions have an energy of about 10 eV, making ion-atom ionization negligible for the increase in Ar ion flux observed in this study.

On the other hand, the heterogeneous charge transfer collision between an Ar atom and He ions is described as:

(7) $$\mathrm{Ar}+{\mathrm{He}}^{+}\overset{}{\to }{\mathrm{Ar}}^{+}+\mathrm{He}.$$

**Figure 10:**
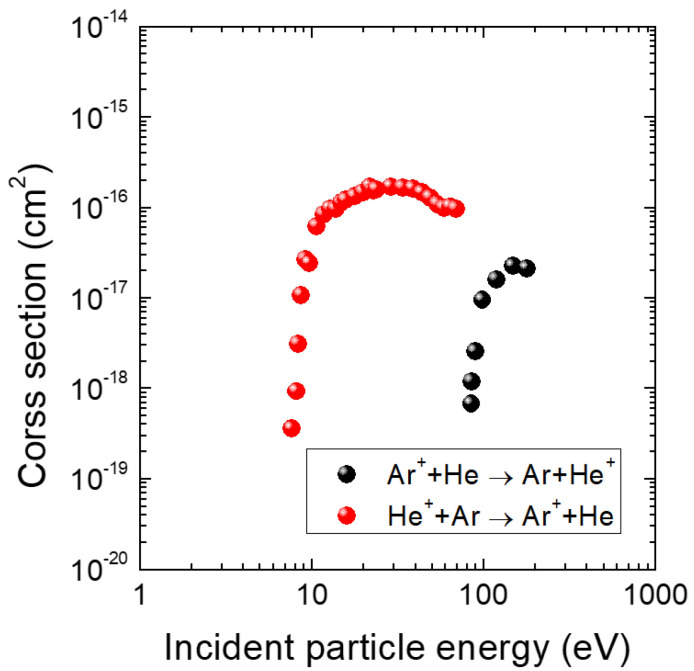
Cross section for heterogeneous charge transfer collisions over incident particle (Ar${}^{+}$ or He${}^{+}$) energy of Ar${}^{+}$-He [37] and Ar-He${}^{+}$ [38].

Figure 10 shows the cross sections of heterogeneous charge transfers [37,38] over incident particle energy. It is worth noting that the threshold energy and the maximum value for this collision are at the energies approximately 9 eV and 12 eV, respectively. Furthermore, the maximum value (∼10${}^{-16}$ cm${}^{2}$) is comparable to electron-impact collisions, such as elastic scattering, excitation, and ionization, which are dominant in an inductively coupled plasma [14]. Conversely, the inverse process of heterogeneous charge transfer between an Ar ion and a He atom (Ar${}^{+}$ + He → Ar + He${}^{+}$) has a significantly higher threshold energy, as shown in Figure 10. Considering the ion energy measured in the study (approximately 10 eV), the inverse process is unlikely to occur. Thus, the dominant collision mechanism responsible for creating Ar ions in the sheath is heterogeneous charge transfer between Ar atoms and He ions.

The heterogeneous charge transfer can be enhanced with increasing chamber pressure. In general, the collision mean free path (λ) is defined as:

(8) $$\lambda =\frac{1}{n_{g}\sigma },$$

where $n_{g}$ is the target gas density and σ is the collision cross section. As the chamber pressure increases, the Ar gas density also increases. The mean free path λ for heterogeneous charge transfer and other collisions becomes shorter, while the sheath width (*s*) can be assumed to be constant due to the fixed $n_{e}$ and $V_{p}$ [14]. This implies that collisions become more frequent in the sheath (λ≪s) with increasing chamber pressure. Regarding the enhanced collision, the decrease in $\Gamma_{{\mathrm{Ar}}^{+}}$ observed in the pure Ar environment, as shown in Figure 8a, may be due to elastic collisions between Ar ions and Ar atoms, which broaden the incident angle of ions and reduce the number of ions reaching the orifice entrance. In the Ar/He environment, the reduction trend follows that of the pure Ar case, but the reduction amount decreases, as shown in Figure 8a,b. In this environment, the two collisions, elastic collision and charge transfer collision, cause the decrease in the reduction amount. The homogeneous elastic collision between Ar atom and Ar ion diminishes due to the decrease in background Ar gas, but the heterogeneous collision between He atom and Ar ion compensates the decrease in the homogeneous elastic collision; since the elastic scattering cross section (Langevin cross section) has a root dependence for the mass of target gas, the heterogeneous collision cross section (He atom–Ar ion) would be similar to the homogeneous collision cross section (Ar atom–Ar ion). As a result, elastic collision effect slightly affects on ion transport in the sheath. Thus, the increase of $\Gamma_{{\mathrm{Ar}}^{+}}$ in Ar/He case implies that heterogeneous charge transfer creates more Ar ions in the sheath with increasing chamber pressure. Therefore, the combined results suggest that heterogeneous charge transfer between Ar atoms and He ions enhances the Ar ion flux on the substrate.

## 5. Conclusions

In this study, we investigated the impact of heterogeneous collisions on the Ar ion flux on the substrate. Our findings demonstrate that the addition of He gas enhances the Ar ion flux, both at a fixed chamber pressure and with increasing pressure at a fixed He addition ratio. While the quasi-neutrality condition suggests a decrease in Ar ion density with the addition of He ions, the observed increase in Ar ion flux implies the creation of Ar ions in the sheath through heterogeneous collisions. Specifically, the heterogeneous charge transfer of Ar atoms with He ions was found to significantly enhance the Ar ion flux on the substrate.

In conclusion, our study provides insight into the role of heterogeneous collisions in influencing the Ar ion flux. The results highlight the importance of considering heterogeneous charge transfer processes in understanding and controlling ion fluxes in plasma systems. 

## Figures and Tables

**Figure A2:**
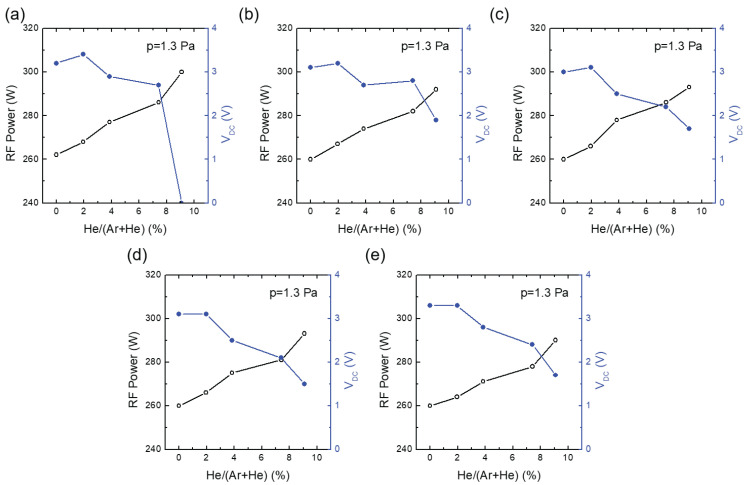
Input RF powers and DC bias voltages (V${}_{\mathrm{DC}}$s) with various additive He ratio at a constant chamber pressure of 1.3 Pa (10 mTorr) at (**a**) 1st, (**b**) 2nd, (**c**) 3rd, (**d**) 4th, and (**e**) 5th trials.

**Figure A3:**
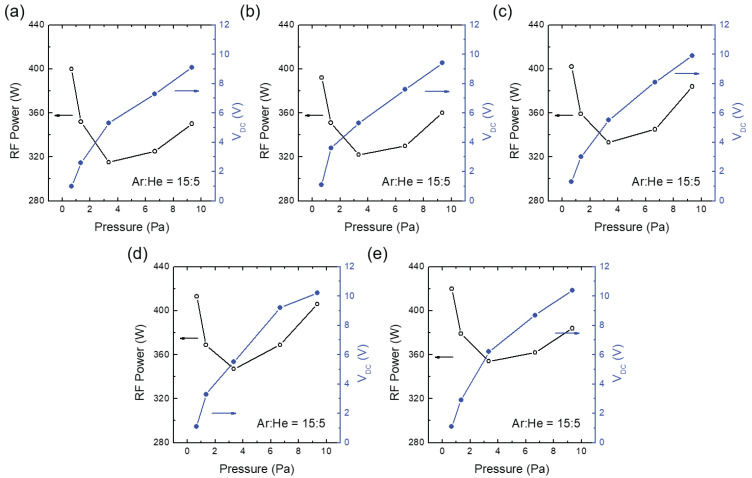
Input RF powers and DC bias voltages (V${}_{\mathrm{DC}}$s) with various chamber pressures with fixed Ar/He ratio (Ar:He = 15:5) at (**a**) 1st, (**b**) 2nd, (**c**) 3rd, (**d**) 4th, and (**e**) 5th trials.

**Figure A4:**
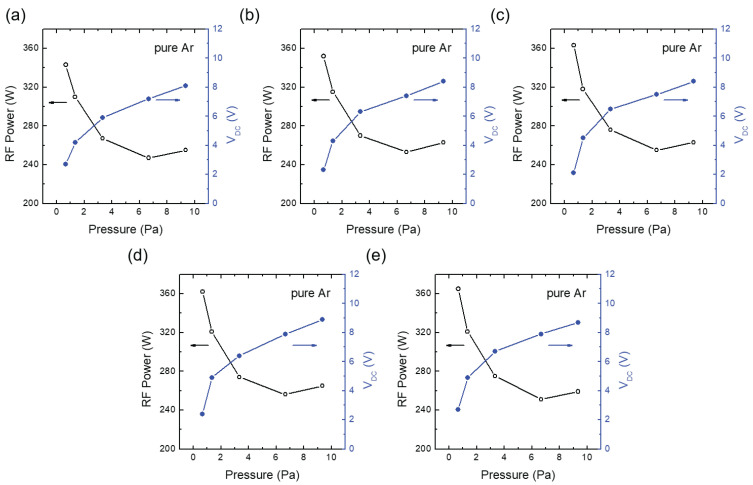
Input RF powers and DC bias voltages (V${}_{\mathrm{DC}}$s) with various chamber pressures in pure Ar environment at (**a**) 1st, (**b**) 2nd, (**c**) 3rd, (**d**) 4th, and (**e**) 5th trials.

## Data Availability

The data presented in this study are available on request from the corresponding author.

## Appendix A

In this section, we list the additional figures used in this study. Figure A1 represents the current–voltage (I–V) curve from the Langmuir probe. Figure A2 represents RF powers and V${}_{\mathrm{DC}}$ voltages to maintain fixed $n_{e}$ and V${}_{p}$ in He addition experiment. Figure A3 and Figure A4 exhibit RF powers and V${}_{\mathrm{DC}}$ voltages to maintain fixed $n_{e}$ and V${}_{p}$ in the pressure variation experiment.
